# Comparative Mitogenomics in *Hyalella* (Amphipoda: Crustacea)

**DOI:** 10.3390/genes12020292

**Published:** 2021-02-19

**Authors:** Francesco Zapelloni, José A. Jurado-Rivera, Damià Jaume, Carlos Juan, Joan Pons

**Affiliations:** 1 Department of Biology, University of the Balearic Islands, Ctra. Valldemossa km 7,5, 07122 Palma, Spain; francesco.zapelloni@uib.eu (F.Z.); jose.jurado@uib.es (J.A.J.-R.); cjuan@uib.es (C.J.); 2 IMEDEA (CSIC-UIB), Mediterranean Institute for Advanced Studies, C/Miquel Marquès 21, 07190 Esporles, Spain; damiajaume@imedea.uib-csic.es

**Keywords:** compensatory mutation, tRNA secondary structure, selection, *Hyalella*, crustaceans

## Abstract

We present the sequencing and comparative analysis of 17 mitochondrial genomes of Nearctic and Neotropical amphipods of the genus *Hyalella*, most from the Andean Altiplano. The mitogenomes obtained comprised the usual 37 gene-set of the metazoan mitochondrial genome showing a gene rearrangement (a reverse transposition and a reversal) between the North and South American *Hyalella* mitogenomes. *Hyalella* mitochondrial genomes show the typical AT-richness and strong nucleotide bias among codon sites and strands of pancrustaceans. Protein-coding sequences are biased towards AT-rich codons, with a preference for leucine and serine amino acids. Numerous base changes (539) were found in tRNA stems, with 103 classified as fully compensatory, 253 hemi-compensatory and the remaining base mismatches and indels. Most compensatory Watson–Crick switches were AU -> GC linked in the same haplotype, whereas most hemi-compensatory changes resulted in wobble GU and a few AC pairs. These results suggest a pairing fitness increase in tRNAs after crossing low fitness valleys. Branch-site level models detected positive selection for several amino acid positions in up to eight mitochondrial genes, with *atp6* and *nad5* as the genes displaying more sites under selection.

## 1. Introduction

In the last decade, the advances in next-generation sequencing–coupled with the improvements in the computer programs for the assembly of short DNA sequences and bioinformatics pipelines for gene annotation–have facilitated the inference of metazoan phylogenies based on mitochondrial protein-coding genes (PCGs). Such phylogenies have proven to be pivotal for reconstructing the evolutionary history of numerous taxonomic groups [1,2,3,4]. Members of the order Amphipoda display the standard metazoan single circular molecule mitogenome with a mean size of about 16 kb, composed of 13 protein-coding genes (PCGs), 22 tRNAs and 2 ribosomal rRNA genes [5,6] plus a non-coding control region [7,8]. The extensive complete or nearly complete mitochondrial sequences obtained to date have shed light on mitogenome evolution. Features such as gene rearrangements, the underlying nucleotide substitution patterns involved in DNA sequence composition and the conservation of rRNA and tRNA secondary structures have emerged [2,9,10,11]. Several of the patterns found in amphipods are common to other invertebrate mitogenomes, including nucleotide composition and AT- and GC-skews biases across coding strands and codon positions plus a higher A+T content at third codon sites, RNA genes and non-coding regions. In addition, NADH dehydrogenase genes (*nad*) of invertebrates display a significant codon usage bias, higher A+T content and sequence divergence compared to cytochrome c oxidase (*cox*) and cytochrome b (*cob*) genes. Invertebrate mitochondrial PCGs also often show non-canonical start codons and truncated stops codons [2,12,13,14]. These distinct features across codon sites and strands need to be carefully considered in phylogenetic reconstructions based on mitochondrial PCG sequences. They have a profound influence on tree topology and branch lengths [15,16].

While many of the mentioned mitochondrial DNA features characterize most metazoans, rearrangements in mitochondrial DNA genes are relatively common when comparing different taxonomic families or even genera within a family [17]. Furthermore, the mitochondrial gene order has been proved to be a reliable source of information concerning phylogenetic relationships [14] such as in cephalopods [18] and snakes [19]. On the other hand, in hymenopterans, gene rearrangements were non-informative since shifts were homoplastic [20]. Although rare, cases of intrageneric gene rearrangements have been reported in different phyla, such as Echinodermata (genus *Cucumaria*) [21], Vertebrata (genus *Odorrana*) [22] and Arthropoda [2,12,23]. Amphipod species of the genera *Metacrangonyx* and *Pseudoniphargus* [2,12] showed different unique gene arrangements, with shifts occurring in different lineages even in the same taxonomic order.

Metazoan mitochondrial tRNAs fold in a cloverleaf structure composed of four arms (acceptor, D-arm, anti-codon and T-arm) except for tRNA-S1 that lost the D-arm in nearly all metazoans [24]. The loss of D- or T-arms in the tRNA secondary structures has been described in several arthropods, e.g., in *Metacrangonyx* amphipods [2], but the lack of both structures (i.e., armless tRNAs) has been noted in spiders and mites [9]. Most mitochondrial tRNAs represent the whole codon family for their associated amino acid (except for leucine and serine that can be attached to two tRNAs from different families), with anticodons under strong evolutionary constraints related to sequence composition [10]. Stems are secondary structures in tRNAs formed by paired sites from distant sequence positions, primarily due to A-U and G-C bonds that provide structural stability. The high strength of the tRNA secondary structures has been related to compensatory mutations preserving the loss of Gibbs free energy in stem structures [25,26]. Two types of compensatory base substitutions (CBCs) are possible; full compensatory base changes (FCBCs), in which both bases of the pair are substituted by another two but conserving the pairing (e.g., AU ↔ GC and GU ↔ UA) and hemi-compensatory base changes (HCBCs), in which just one base of the pairing is substituted (e.g., AU ↔ GU, GC ↔ GU). The pattern of compensatory substitutions in invertebrate mitochondrial tRNA stems is poorly known.

Due to their role in energy metabolism, mitochondrial oxidative phosphorylation system genes are primarily under background purifying selection [15,27]. However, distinct environmental conditions can impose selective pressure on the mitochondrial respiratory chain genes. Positive selection was proposed for mitochondrial PCGs of three decapod genera adapted to the anchialine (aquatic subterranean) environment [28]. Similar findings were reported in the crustacean family Alvinocaridae living in hydrothermal vents [29].

The species of the genus *Hyalella* Smith, 1874 constitute the only epigean amphipods that occur in freshwater environments of the Americas [30]. The genus comprises 80 recognized species, with a considerable presence (28 species) in the Titicaca, an ancient lake located in the Andean high plateau of the central Andes at an elevation of 3806 m. Thus, *Hyalella* species occur in both high- and low-altitude freshwater environments, each habitat encompassing contrasting differences in oxygen concentration and UV-radiation, factors that may potentially lead to environment-specific adaptations. These habitat differences make the genus *Hyalella* an interesting model for studying natural selection acting in the mitogenome that may have resulted in adaptive divergence. The phylogenetic relationships of South American *Hyalella*, with particular focus on taxa of the Andean Altiplano, have been examined using mitochondrial cytochrome oxidase subunit 1 [31,32] and nuclear ribosomal [32] or histone H3 sequences [31]. Molecular species delimitation criteria revealed 36 molecular operational taxonomic units within the South American *Hyalella* species sampled. Eleven of these taxa could be endemic to Lake Titicaca and the nearby lakes in the northern Andean Altiplano [31]. Recently, a phylogenetic analysis based on mitochondrial PCGs, nuclear ribosomal and single-copy nuclear gene sequences showed that the Andean Altiplano was colonized by South American lineages in the Miocene, with a more recent diversification from late Pliocene to early Pleistocene [33].

Here, we analyze in more detail the genome organization and evolution of 16 *Hyalella* mitochondrial DNA sequences obtained as complete or nearly complete mitogenomes. The main aims of this work are to (i) perform a comparative analysis with a North American representative of the genus (*H. azteca*), (ii) provide insights in the evolution of tRNA molecules through the analysis of compensatory mutations and (iii) explore patterns of positive selection in the PCGs across *Hyalella* species.

## 2. Materials and Methods

### 2.1. Sampling

Localities and GenBank accession numbers of the 16 South American *Hyalella* species studied here are listed in Table S1. Samples were collected from the lakeshore or a boat by either using a hand-held plankton net or a small dredge. All samples were then preserved in ethanol [34]. For comparison purposes and selection analyses, the mitogenome of *H. azteca* and mitochondrial genomes of twenty amphipod species were downloaded from GenBank (Table S1).

### 2.2. DNA Extraction, Mitogenome Assembly and Annotation

Molecular procedures are fully described elsewhere [33]. Briefly, genomic DNA was purified from single specimens using the Qiagen DNeasy Blood & Tissue kit (Qiagen, Hilden, Germany) following the manufacturer’s instructions. The Hyper Library construction kit from Kapa Biosystems (Wilmington, MA, USA) was used to construct individual shotgun genomic libraries from 100 to 500 ng of genomic DNA TruSeq adapters (Illumina, San Diego, CA, USA). We pooled libraries in equimolar concentrations that were pair-end sequenced (2 × 150 bp) in a single lane of Illumina HiSeq2500 (Illumina, San Diego, CA, USA). Adapter sequences and low-quality bases (< Q30) were removed in Trimmomatic v0.33 [35], and both paired and unpaired clean reads were assembled in SPAdes (v3.13) using three kmers (21, 35 and 47 nucleotides) to maximize assembly yield [34]. Mitochondrial contigs were filtered out using BLASTn (*e*-value −30) against the mitogenome of *H. lucifugax* (GenBank accession number LT594767) as a reference. We assessed the completion of the mitochondrial contigs using the script circularizationCheck.py (mitoMaker) [36], and incomplete contigs were extended by iterative mapping of reads in GENEIOUS v11.1.5 [37]. The mitogenomes were annotated using MITOS2 [38] and genes manually curated in GENEIOUS, particularly at 5′ and 3′ ends.

### 2.3. Composition Analyses and Gene Order

Nucleotide, amino acid, and codon compositional analyses at both complete mitogenome and single gene sequences were performed using the Python script from Narakusumo et al. [39] and plotted with the Python package matplotlib [40] and the R [41] packages ggplot [42] and heatmap [43]. We used CREx [44] to analyze mtDNA gene rearrangements and the molecular mechanism driving them. The AT- and GC-skews were calculated using the formulas: (A-T)/(A+T) and (G-C)/(G+C), respectively [45]. Codon usages (effective number of codons, ENC; and measure independent of length and composition, MILC) and GC content of third codon sites of fourfold degenerated amino acids were estimated in INCA v2.1 [46].

### 2.4. Analyses of the Secondary Structure of tRNAs

Prediction of secondary structures of tRNAs was carried out using Aragorn v1.2.38 [47], and full compensatory base changes (FCBCs) occurrence among *Hyalella* species was analyzed using 4Sale v1.7.1 [48]. A hierarchical cluster analysis of FCBC occurrence was carried out using the R package heatmap2. The resulting cladogram was compared with the *Hyalella* mtDNA phylogeny through a tanglegram analysis in the R package dendextend [49]. We also used dendextend to compute the Baker γ index [50], an index of similarity between two trees of hierarchical clustering (i.e., dendrograms). Ancestral reconstruction of tRNA sequences aligned in MUSCLE v3.8.1551 [51], with minor modifications in D- and T-loops, was performed in prank v.100311 (commands -showanc -showevents -keep). We used the most common secondary structure for each alignment (50% majority rule), including gaps, in D- and T-arms (e.g., tRNA-A), and set acceptor and anti-codon arms with seven and five pairs, respectively, despite the presence of mismatches. The Bayesian tree topology estimated elsewhere in BEAST [52] for the 13 PCGs was used as a topological constraint to estimate ancestral sequences [33] after pruning non-included taxa.

### 2.5. Phylogenetic Inference and Selection Analyses

**Figure 1:**
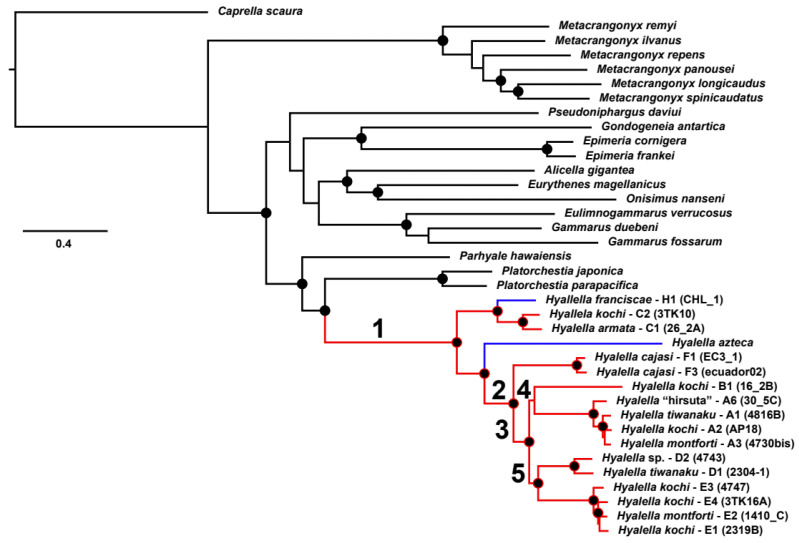
Maximum likelihood phylogeny. Phylogeny based on the concatenated 13 mitochondrial protein-coding genes (PCGs) of *Hyalella* and other amphipod genera (see Table S1 for details). Nodes with bootstrap support ≥ 98 are indicated with black circles. The *Hyalella* lineage is indicated with red branches, except for the taxa dwelling at low altitude in blue. Numbers on nodes indicate branches individually used as foreground for selection analyses.

The mitochondrial PCGs from the *Hyalella* species plus a dataset composed of twenty species belonging to eleven amphipod genera (Table S1) were translated into proteins, aligned using MUSCLE [51] and back-translated to DNA. The resulting single-gene alignments were concatenated with Phyutility [53] and used as input in IQTREE v.2.1.10 [54,55] to estimate the best partitioning scheme plus evolutionary models and to build a maximum likelihood tree. The phylogenetic tree obtained (Figure 1) was used in PAML v4.7 [56] with non-synonymous/synonymous substitutions ratios (*ω* = *d*_N_/*d*_S_) estimated using the codon-based maximum likelihood method. We aimed to explore signals of adaptive evolution in mitochondrial protein-coding genes that could be related to environmental adaptations of freshwater amphipods, particularly adaptation to high-altitude hypoxic conditions in the Altiplano. We applied the branch-site model A (also known as test 2) to test for positive selection. This test can detect episodic selection affecting particular codons in a protein-coding gene along predefined branches on a tree, with selection measured by the *d*_N_/*d*_S_ ratio and positive selection accepted when *ω* > 1. For this analysis, branches on the phylogeny are divided a priori into foreground and background lineages, with the former defined as the one that may have experienced positive selection. Alternative branches in the *Hyalella* phylogenetic tree were specified as the foreground to test for positive selection with their significance evaluated through a likelihood ratio test (LRT). Branch 1 included all *Hyalella* to the respective background (remaining) clades. Branch 2 corresponded to the *Hyalella* South American clades A, B, D, E and F occurring at altitudes higher than 3800 m as foreground to the North American *H. azteca* plus South American clade C (the latter including two species dwelling at low altitude, namely *H. franciscae* and *H. azteca*). This contrast was designed to test if hypoxic high-altitude adaptations match with adaptive evolution of mitochondrial electron transport chain and oxidative phosphorylation genes. Additionally, branches 3, 4 and 5 corresponded to further internal nodes in the South American *Hyalella* phylogeny, all of them composed of high-altitude species. The branch-site model A likelihood was compared with the corresponding to the null hypothesis (H0, i.e., branch-site model with the parameter ω = 1 fixed as all mutations neutral). We also applied the site model M8 vs. M8a to identify the probability of a site to be under selection [57], using a ω value able to vary among amino acid positions. Positive selection was also analyzed using the program TREESAAP [58]. This analysis compares specific aminoacidic property changes in non-synonymous residues across a predefined phylogeny and identifies the ones most likely influenced by positive selection [59]. The TREESAAP results were plotted using the package ggplot2 [42] in R [41].

## 3. Results

### 3.1. Genome Organization

**Figure 2:**
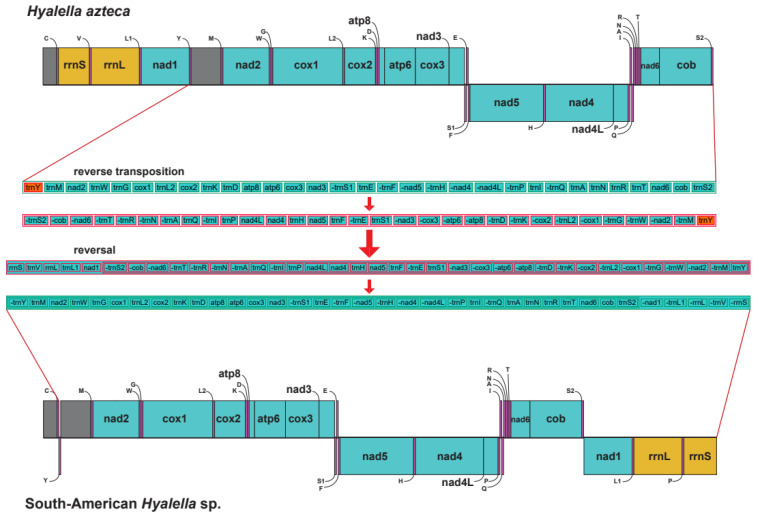
*Hyalella* mitochondrial genome maps. Gene order comparison between the mitogenomes of South American *Hyalella* species and the North American *H. azteca*. Genes located above the central line correspond to genes coded on the plus-strand, whereas those coded on the minus-strand appear below. Blocks in grey correspond to non-coding segments attributed to control regions. Shifts accounting for the observed differences between the two genome arrangements obtained with CREx are shown, with steps symbolized by arrows.

Five out of the 16 mitogenomes of South American species studied here were complete, namely samples *H. tiwanaku* codes 2304(1) and 4816-B, *H.* “hirsuta” code 30_5C, *H. cajasi* code EC3_1 and *H. montforti* code 4730_bis. The remaining ones lacked one or two non-coding segments corresponding to the control regions, and for most of them, we could not recover *trnC*, *trnM* and *trnY* genes since they are adjacent to repetitive control regions. *H. kochi* (code 16_2B) was the exception since it was highly fragmented, due to low or inexistent coverage in many regions. Thus, in this species, most of the tRNAs and the two rDNA genes could not be retrieved. Hence, its tRNA sequences were not included in the analyses (Table S2). Mitogenome length ranged from 13,972 to 15,204 bp (average 14,434; SD ± 440.15 bp). Complete mitochondrial genomes included the typical set of 37 genes found in metazoans (13 PCGs, 22 tRNAs and 2 rDNA genes) plus two control regions. Twenty-four genes were located in the plus-strand and thirteen in the minus one in South American *Hyalella* mitogenomes. In comparison, the mitogenome of North American *H. azteca* showed only eight genes coded in the minus-strand (Figure 2). South American species also showed a conserved gene order that differed from the North American counterpart *H. azteca* (Figure 2). The CREx analysis performed comparing the mitochondrial genome organization of *H. azteca* and the South American species showed that gene block composed of *cox1*, *trnL2*, *cox2*, *trnK*, *trnD*, *atp8*, *atp6* and *cox3* was conserved between the ancestral pancrustacean and the *Hyalella* species, but multiple reorganization events seem to have happened throughout the genome. Three transpositions (*trnN*, the block *trnM*, *nad2* and *trnW* and *trnG*), two feature reversals (*trnS1* and *trnC*) and a tandem duplication with subsequent random gene loss (the block of the ancestral pancrustacean from *trnA* to *trnQ*) (Figure S1) can be deduced. The CREx comparison of the South American and North American *Hyalella* mitogenomes showed two major shifts (Figure 2), namely a reverse transposition (i.e., transposition from different strands) of the gene block flanked by *trnY* and *trnS2*, and a second reversal of most of the genome except the *trnC* gene.

### 3.2. Base Composition and AT- and GC-Skews

**Figure 3:**
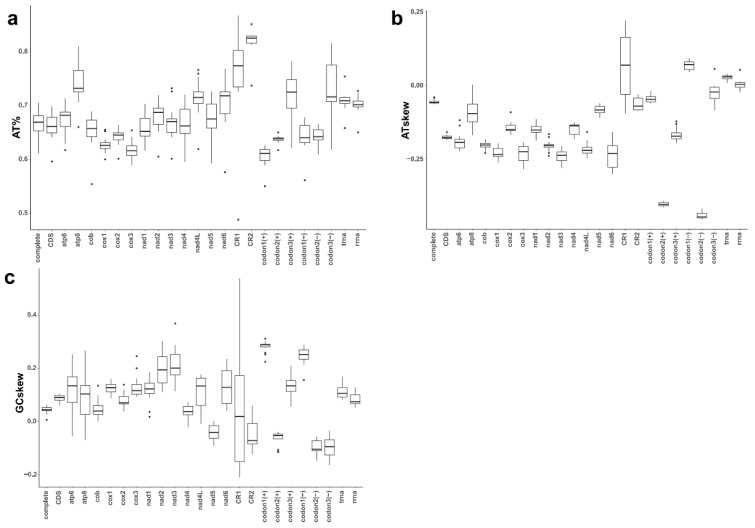
Mitochondrial nucleotide composition in *Hyalella*. Box plots showing values of nucleotide composition (A+T percentage) (**a**), AT-skew (**b**) and GC-skew (**c**) across *Hyalella* mitogenomes and indicated as complete, genes, codons, partitions and strands.

The A+T content of *Hyalella* mitogenomes ranged from 61.1% to 70.5% (average 66.9%, SD ± 2.3%) (Figure 3a). The AT-skew covered a range between −0.042 and −0.066 (average −0.058; standard deviation 0.007) whereas the GC-skew was between −0.042 and 0.214 (average 0.043; standard deviation ± 0.014) (Figure 3b,c). The *atp8* gene showed the highest standard deviation in A+T%, AT- and GC-skews, with values of 0.03, 0.049 and 0.104, respectively. This fact may be due to its short length since this gene also displayed an extremely high divergence across species. The two non-coding regions (CR1 and CR2) had the highest A+T frequencies, with CR1 showing the highest variability in the three base composition characteristics analyzed. Base composition and AT- and GC-skews showed marked differences when analyzed by codon position and coding strand (Figure 3a–c). The average A+T frequency at the third codon positions (72.3%) was higher than in the other two codon sites (first positions 60.6%, second positions 63.7%) though they were divergent across species. The percentage of A+T content in the first and second codon sites of the minus-strand was slightly higher than in the plus one (Figure 3a). The average AT-skew in the first codon positions in the plus and minus-strands were −0.047 and 0.067, respectively (Figure 3b). The second codon positions showed lower AT-skew values, −0.405 in the plus and −0.442 in the minus-strand. However, larger AT-skew differences were observed between plus and minus-strands at the third codon sites (plus-strand: −0.169, minus-strand −0.025) (Figure 3). Average GC-skew of the first (plus 0.282, minus 0.246) and the second codon sites were similar (plus −0.063, minus −0.095), while there were marked differences regarding third codon positions (plus 0.135, minus −0.101) (Figure 3c). The patterns found in rDNA and tRNA sequences showed higher A+T content and AT-skews but similar GC-skews compared to other genes and regions (Figure 3). The differences between stem and loop regions in tRNAs were larger regarding A+T content (66.2% vs. 77.6%), and AT-skews (−0.068 vs. 0.135) but smaller for GC-skews (0.098 vs. 0.179), i.e., stem sequences were relatively Ts and Gs-rich, and loops As and Gs-rich (Table S3).

### 3.3. Start and Stop Codons, Amino Acid Composition and Codon Usage

We found that most mitochondrial PCGs start with the canonical codon triplets ATG and ATA, but several exceptions displayed the alternative ATT and ATC codons (Table S4). Several non-canonical start codons were clade-specific. For instance, the two mitogenomes of the Ecuadorian species *H. cajasi* showed GTG as the initiation codon for the genes *cox2* and *atp8*. The gene *nad5* starts with TTG in all *Hyalella* taxa with the exception of members of Clade C [33] (*H. armata* code 26_2A; *H. kochi* code 3TK10 and *H. franciscae* code CHL-1) that have CTG instead and in the North American *H. azteca* (ATA). The non-canonical GTA start codon was identified in the *nad1* gene of the Ecuadorian *H. cajasi* and the Chilean *H. franciscae*. Stop codons generally were the canonical TAA and TAG, but some genes showed truncated stop codons in most species (e.g., *nad2*, *nad4* and *nad5* genes).

Some amino acids were more frequent in the mitochondrial PCGs, such as leucine (~20%), serine (~15%), phenylalanine and valine (~10% each). In contrast, others were rarely found (cysteine, aspartic acid, histidine, glutamine, arginine and tryptophan, with less than 2% each) with minor differences across species and between coding strand (Figure S2). For each amino acid, the codons with higher A+T richness are used frequently, e.g., ATA (M), AAA (K), TTT (F), CAA (Q), TGA (W), TAT (Y), AAT (N), ATT (I) and TTA (L) (Figure S3). Codon usage (estimated using ENC values) and GC content of third codon sites of fourfold degenerated amino acids (GC3) is positively correlated (i.e., higher A+T content is associated with higher codon usage bias; see Figure S4). The analysis of codon usage based on MILC values, unbiased against length and nucleotide composition [46], corroborated ENC results and indicated that codon usage was not strongly biased. MILC values around 0.5 suggest that all codons are equally used (ENC 62) whereas higher numbers 1.9 indicate the usage of few codons (ENC 26). The codon bias was less pronounced on the genes encoded on the minus-strand (Figure S4).

### 3.4. tRNA Secondary Structures and Compensatory Mutations

**Figure 4:**
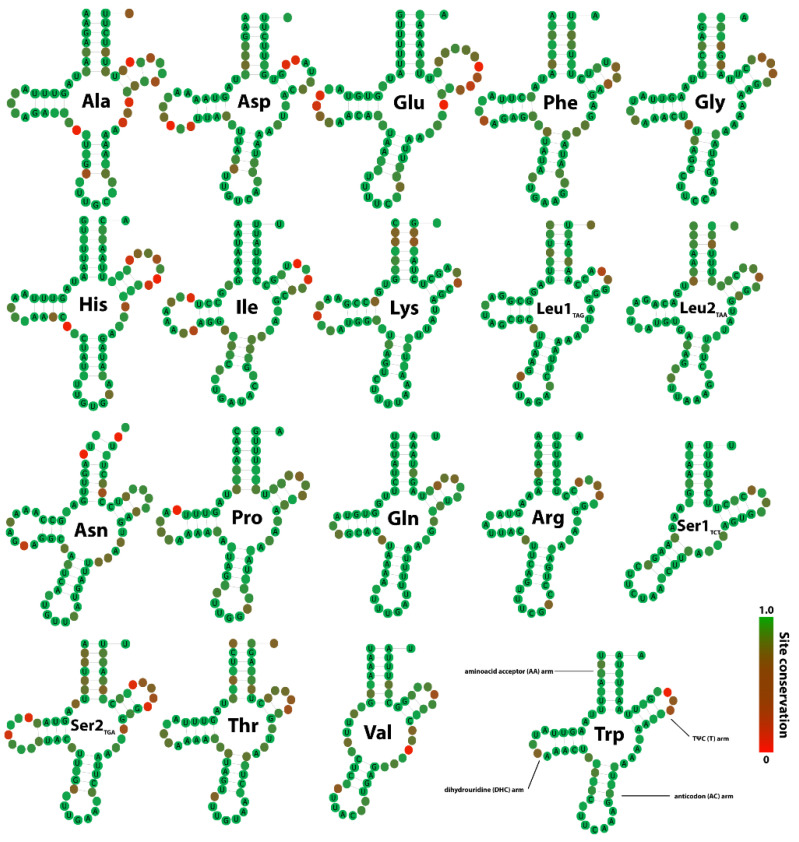
Mitochondrial tRNAs structures. Secondary structures predicted for the 19 studied Table 0. means Scheme 539. and 738 changes were found in stems and loops, respectively (Table S6). Some tRNAs’ stems showed a higher number of substitutions (e.g., S2 51, F 48, and T 41 and A 40) whereas others exhibited values lower than average (e.g., E 16, R 16, L1 17 and S1 17). Stems included some indels (13) since analyses are based on comparisons to a single secondary structure (majority-rule consensus) per alignment and D- and T-stem sequences were different in length. The analysis in loops reconstructed more nucleotide substitutions and indels (96) than those found in stems with some tRNAs displaying more changes (S2 59, H 58, E 57 and V 54) than others (K 17, L2 23, R 27 and W 27). The most frequent substitutions were transitions A -> G, T -> C, G -> A and C -> T in stems and loops but the latter also showed high frequencies A -> T, T -> A, T -> C and C -> T (Table S6).

**Figure 5:**
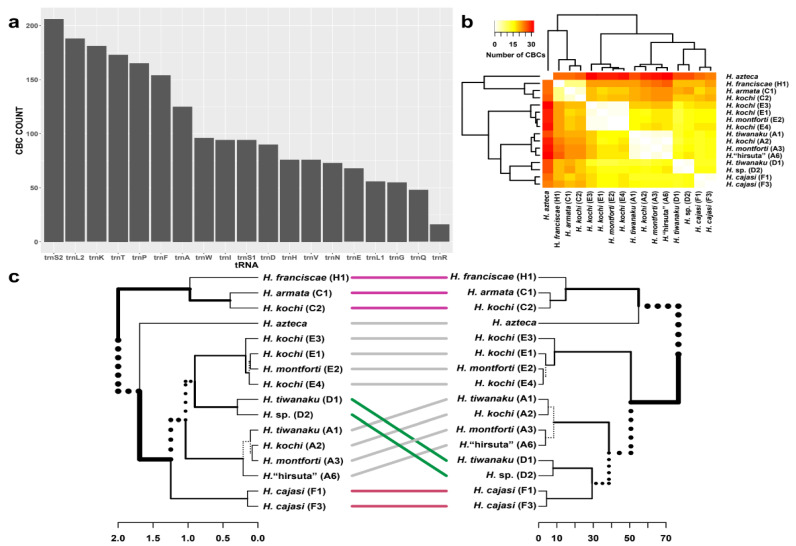
Analysis of the compensatory mutations. Bar plot showing the number of compensatory base changes (y-axis) in *Hyalella* tRNAs (x-axis) (**a**). Heatmap summarizing results of the hierarchical clustering analysis. Warmer colors (red–orange) indicate a higher number of compensatory base changes between samples (**b**). Tanglegram confronting the subtree of the genus *Hyalella* from Figure 1 (left) and the dendrogram of the hierarchical clustering analysis (right) (**c**). Dotted lines indicate branches differing in the two trees, while thick lines and in colors show similitudes between them.

We identified the whole set of 22 tRNA genes in the complete *Hyalella* mitogenomes except for *H. kochi* code 16_2B. Three tRNAs (C, Y and M) and one mitogenome (*H. kochi*, code 16_2B) were excluded from the analysis of compensatory substitutions to minimize missing data, and the North American species *H. azteca* was included as a putative outgroup. Secondary structure folding matched the typical cloverleaf pattern (Figure 4) except for tRNA-V and tRNA-S1 that lacked the D-arm. Anti-codon sequences were conserved across species (Table S5). The total number of compensatory mutations across all pairwise comparisons between *Hyalella* species ranged from 206 in tRNA-S2 to 16 in tRNA-R (Figure 5a). A hierarchical cluster analysis was performed to obtain a dendrogram from the set of compensatory mutation dissimilarities (Figure 5c). The Baker index obtained by correlating the cluster analysis of compensatory mutations and the *Hyalella* phylogenetic tree based on PCGs was 0.862.

**Figure 6:**
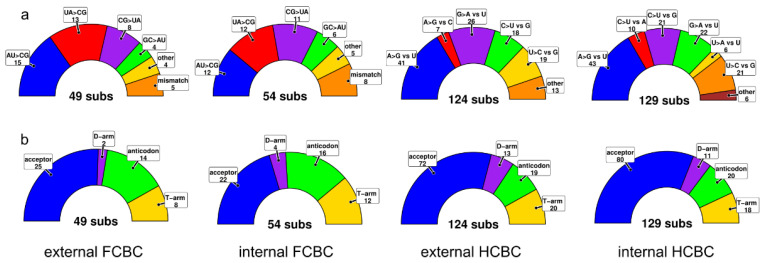
Full compensatory base changes (FCBCs) and hemi-compensatory base changes (HCBCs) in tRNAs. Type of nucleotide substitutions in full compensatory base changes and hemi-compensatory base changes (**a**) and number of nt switches per tRNA arm (**b**) deduced to be on internal and external branches of the phylogenetic tree.

We found 49 FCBCs in stems (i.e., equivalent to 98 changes, as both bases of the pair are affected) in external branches or nodes (substitutions in extant species) and 54 in internal branches/nodes (substitutions reconstructed in ancestral sequences). Regarding the number of HCBCs, 124 changes were found in terminal branches, and 129 substitutions were deduced in the internal ones (Figure 6). Most of the compensatory nucleotide changes were found in the acceptor’s arm, followed by anti-codon, T- and finally D-arms, irrespective of being in internal or external branches (Figure 6). The majority of FCBCs in external/internal branches were Watson–Crick switches AU -> GC (15/12), and GC -> AU (13/12), CG -> UA (8/11) and GC -> AU (4/6). Other FCBCs were found in low frequencies (e.g., UA -> AU) or were mismatches converted to matches and vice-versa. The majority of HCBCs in external branches were A -> G vs. U (41) and G -> A vs. U (26) that also included a few non-Watson–Crick pairings such as U -> C vs. G (19) and C -> U vs. G (18) whereas other changes added up to 20 substitutions. HCBCs in internal branches retrieved a similar pattern (Figure 6).

### 3.5. Positive Selection Analyses in Protein-Coding Genes

Five branches of the maximum likelihood amphipod mitogenomic tree obtained (Figure 1) were used as a foreground for the branch-site selection model analysis in CodeML. The analysis using Branch 1 as foreground (the common ancestor of all *Hyalella*) reported eight genes with codon positions under positive selection with statistically significant LRTs (see Table S7; *atp6*, *atp8*, *cob*, *cox1*, *cox2*, *nad4*, *nad5* and *nad6*). According to the Bayes empirical inference [28], the numbers of sites under selection were: 21 in *atp6*, 1 in *cox1*, 5 in *cox2*, 6 in *nad4*, 15 in *nad5* and 2 in *nad6* (Table S7). For two of these genes (*atp8* and *cob*) the program could not identify sites under selection. The genes under selection using Branch 2 as foreground were *atp6* (2 sites), *nad1* (1), *nad2* (3), *nad4* (4) and *nad5* (2) (Table S7. We found only *nad2* with positions under positive selection when implementing Branch 3 as foreground. No evidence of positive selection was found using the internal *Hyalella* branches. Site model M8 only reported selection in codons 431 and 437 of *nad5*, with posterior probability values lower than 0.95 in both cases.

**Figure 7:**
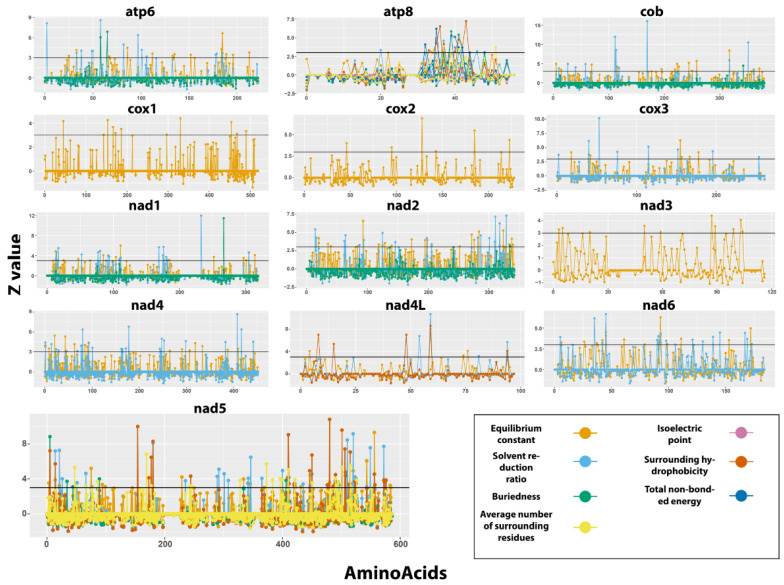
Selection on structural and biochemical amino acid properties of *Hyalella* mitochondrial PCGs. The x- and y-axis correspond to amino acid sites and Z-score values per site, respectively. The *Z*-score was calculated for the two most radical magnitude categories considered in TreeSAAP (7 and 8). Colors indicate the different physicochemical characteristics deduced to be under positive selection, as shown in the figure. The horizontal line corresponds to the minimum *Z*-score value for positive selection to be considered significant at a particular site.

The program TREESAAP classifies the radical amino acid changes into eight magnitude categories [58], with higher categories indicating substantial changes in the amino acid physicochemical property. The property with a Z-score higher than 3.09 for the most radical categories (7 and 8) was selected to be under a positive selection (Figure 7). The equilibrium constant (ionization of COOH) (category 8) is a chemical property that seems to be under positive selection for all the 13 PCGs. Ten genes (*atp6*, *atp8*, *cob*, *cox3*, *nad1*, *nad2*, *nad4*, *nad4L*, *nad5* and *nad6*) are under positive selection for the solvent reduction ratio (category 7). Buriedness (categories 7 and 8) are under selection for *atp6*, *atp8*, *cob*, *nad1*, *nad2* and *nad5*. The genes *atp8*, *nad4L* and *nad5* are under selection for four other properties, namely an average number of surrounding residues (category 8, *atp8* and *nad5*), isoelectric point (category 7 and 8, *atp8*), surrounding hydrophobicity (category 8) and total non-bonded energy (category 7, *atp8*).

## 4. Discussion

### 4.1. Hyalella mtDNA Sequence Features

The complete mitogenomes of *Hyalella* obtained were of similar length to those described in other amphipods (14.9–15.2 kb) [2,12,60,61]. The high A+T content found in *Hyalella* mtDNA sequences (67.2%) is in the middle range of the A+T content of other crustacean and invertebrate mitogenomes [12,62], but lower than in other amphipods [12,63]. Although there are several exceptions (e.g., genus *Caprella* and metacrangonyctid taxa), the control region in most amphipods is located between *rrnS* and *trnY* genes (both in the minus-strand) [2]. The South American *Hyalella* exhibit a control region flanked by *rrnS* (minus-strand) and *trnC* (plus-strand), while in the North American *H. azteca* this region is located between *trnS2* and *trnC* (both in the plus-strand). The positioning of the control region in *H. azteca* appears to be specific, as the presence of a second putative control region in the South American *Hyalella* clade. The gene order common to all South American species supports the sister relationship to *H. azteca* as the most plausible hypothesis [33]. Additional mitogenome sequences from other North American species are needed to fully resolve both the reciprocal monophyly of South and North American species and if there are clade-specific mtDNA gene orders.

A pronounced strand bias characterizes metazoan mitogenomes in terms of nucleotide composition [17]. Malacostracan crustaceans typically show negative GC-skew (i.e., excess of C) in the plus-strand genes and a positive GC-skew (i.e., higher Gs) in the genes encoded on the minus-strand due to differential selective pressure associated with DNA replication and transcription [64]. However, Isopoda and the amphipod family Metacrangonyctidae have been reported to be exceptions [2] as are the *Hyalella* species, since all mitogenomes studied here show a positive GC-skew in all PCGs from both strands, except for *nad5*. The cob gene is coded on the plus-strand in most pancrustaceans, including *Pseudoniphagus* amphipods [12] and *Hyalella*. Nevertheless, in Metacrangonyctidae this gene appears to have shifted to the minus-strand and also shows a negative GC-skew (~ −0.25) [64]. On the other hand, AT-skew values in PCGs are negative across the crustaceans studied so far, including the *Hyalella* and amphipod groups [2,12]. Ribosomal coding genes also showed very similar trends across amphipod genera, with no bias in AT-skews (i.e., equal A and T frequencies) and positive GC-skews.

*Hyalella* mitogenomes also showed differences among first, second and third codon sites as previously described in most invertebrates [2,12,15,65]. The typical pattern is that third codon sites display a markedly higher A+T content than first and second codon positions [2,14,65]. In *Hyalella*, AT-skews also vary among the three codon sites, with first and third codon sites with nearly no differences in A and T frequencies (AT-skew values close to zero) but high negative values (~−0.4) in second codon sites, with minor differences in genes encoded in plus and minus-strands. GC-skews in *Hyalella* also showed differences across the three codon sites, with positive values (~0.2) in first codon sites, negative, although nearly with no bias on second codon sites, and finally, slightly negative values for third codon sites with slight variations between coding strands. These patterns and values were similar to those described in *Metacrangonyx* [2] and *Pseudoniphargus* [12] except for GC-skews values of third codon sites in Metacrangonyctidae species that showed most genes coded in the plus-strand showing small positive values, while showing negative values in the minus-strand [45].

The presence of non-canonical start codons (mostly ATN and NTR codons) and truncated stop codons (T and TA) in several *Hyalella* PCGs confirms that this is a relatively common feature of invertebrate mitochondrial genomes [2,10,66,67,68]. *Hyalella* mitochondrial PCGs are A+T-rich, with no strand differences as having been described in most pancrustaceans [3,18,69]. However, this AT-richness has a profound impact on the codon usage since the reduction of G+C content also lessens the number of codons implemented in proteins with slightly stronger bias in PCGs encoded in the plus-strand (Figure S3). This pattern has been extensively described in many invertebrate mitochondrial studies [2,67], with the exception of *Pseudoniphargus* [12]. As it has been previously described in Metacrangonyctidae [2], *Pseudoniphargus* [12] and other crustaceans [67,68,70], insects [71,72,73] and spiders [9], mitochondrial proteins of *Hyalella* are particularly rich in serine and leucine, whereas other amino acids are at minor frequencies. The amino acid frequencies are quite similar regardless of the coding strand of their gene, with slight variation across *Hyalella* species. However, significant differences were found among the amino acids more frequently used, as in *Metracrangonyx* amphipods [2]. The amino acid pattern in *Hyalella* suggests that invertebrate mitochondrial genetic code is under selection constraints to minimize the effect of translational misread errors of single nucleotide substitutions [74]. The optimal assignment of amino acids to codons is attained by mapping codons differing by a single nucleotide to the same amino acid or a chemically related one. Additionally, amino acids with simple chemical structure and smaller size tend to have more codons assigned, such as serine since they are less likely to be rejected by selection [74,75]. Moreover, both leucine and serine amino acids have two tRNAs due to an ancestral duplication that presumably occurred in a metazoan ancestor about 600 Mya [76]. Furthermore, the two anti-codons for each amino acid are quite different, so any mutation in a codon originally specifying another amino acid has more probabilities of being recognized by a serine or leucine tRNA [75]. These factors could explain why in invertebrate species, serine and leucine are so frequent in mitochondrial-coded proteins.

### 4.2. Mitochondrial Gene Arrangement in Hyalella

The South American *Hyalella* mitogenomes reported here match the mitochondrial ancestral pancrustacean arrangement characterized by the location of the *trnL2* (UUR) gene between *cox1* and *cox2* [13,66]. The conserved block of *nad1*, *trnL1*, *rrnL*, *trnV* and *rrnS* genes is retained in the mitochondrial genomes of *Hyalella* species. The *Hyalella* gene block *cox1*, *trnL2*, *cox2*, *trnK*, *trnD*, *atp8*, *atp6* and *cox3* is also found in most amphipod mitogenomes [1,2,60,63,77] (Figure S5), and the same applies to the gene block encoded at the minus-strand *nad5*, *nad4*, *nad4L* and *trnH* [2]. Interestingly, the block *trnA*, *trnS1*, *trnN*, *trnE* and *trnR* commonly reported from most amphipods [77] shows a different arrangement in *Hyalella.* This gene order matches that of the related talitrid species *Parhyale hawaiensis* [60], which could be indicative of an ancestral clade-specific gene rearrangement affecting the families Hyalidae and Hyalellidae. On the other hand, the *Hyalella* spp. mitochondrial genomes show several genus-specific features such as the loss of *trnL2* between *cox1* and *cox2* and the transposition of the gene *nad3* (Figure S5). Our analyses also revealed the occurrence of a complex mitogenome rearrangement within South and North American *Hyalella* taxa involving a reversal transposition followed by a secondary reversal. Mitochondrial gene rearrangements between species of the same genus are a rare phenomenon in arthropods, and to our knowledge, the *Hyalella* would represent the second case reported in amphipods [12]. We interpret that these differences derive from an ancestral divergence between the *Hyalella* lineages inhabiting the different landmasses of the American continent [32,33].

### 4.3. Secondary Structures and Compensatory Base Changes in tRNAs

*Hyalella* tRNAs are folded in the typical cloverleaf secondary structure, with tRNA-S1 and tRNA-V lacking the D-arm. The absence of D-arm in tRNA-V was previously described in the amphipod species of the genus *Pseudoniphargus* and Metancrangonyctidae family. However, they also lacked the T-arm in tRNA-Q and D-arm in tRNA-R, tRNA-S1 and tRNA-S2, respectively. These results are consistent with the loss of D-arm in tRNA-S1 in most metazoan [24] and the absence of the same arm in tRNA-V in other amphipods [2,12,77].

Stems in RNAs consist of nucleotide sequences that form base-pairings with complementary regions within the same strand. Ribosomal tRNA secondary structures are subject to evolutionary constraints as nucleotide changes dismantling stable base-pairs are potentially deleterious. Only 6 of the 16 possible base-pairings are stable (the Watson–Crick pairs AU, UA, GC, CG and the “wobble” pairs GU and UG). Thus, to maintain the RNA structure, a mutation in a stem must be compensated by a nucleotide matching the pair’s mutated base. The analysis of the nucleotide composition and nucleotide substitution patterns of *Hyalella* tRNAs showed differences between stems and loops as described previously [2,10,25], possibly reflecting distinct evolutionary patterns of the two structures. Generally, A+T frequencies in loops were about 10–15% higher than in stems, perhaps related to the higher frequencies of nucleotide substitutions U -> A and A -> U that are not frequent in stems. Moreover, in *Hyalella* tRNAs, AT-skews were slightly lower in stems than in loops, so the latter have a slight bias towards including more Ts (Us), able to pair either with A or G, making a Watson–Crick and a wobble pairing, respectively. 

Models for phylogenetic inference have been developed for virus RNA (doublet substitution models in stems implementing compensatory mutations in base-pairing) as secondary structures are likely to violate the assumption that nucleotide substitutions occur independently among sites [78]. Analysis of compensatory base changes has been performed in mitochondrial tRNAs in a few cases (e.g., [10,25,26,79]). Two models have been postulated to explain the compensatory evolution in tRNAs in a fitness landscape: the “continuous fitness ridges” and the “isolated fitness peaks” or “valley hypothesis” [25,26]. In the first model, Watson–Crick switches from AU to GC or vice-versa occur independently and are not expected to cluster on the phylogenetic tree. The AC and GU pairs are considered intermediate stages with weaker pairings and hence with lower fitness. This model further splits into a “flat continuous fitness ridges” where AU, GU and GC pairings possess high fitness and AC an extremely low fitness, and “ascending continuous fitness ridges” where GC represents the highest fitness, AU and GU intermediate values and AC the lowest one. The isolated fitness peaks hypothesis suggests that an AU ↔ GC switch is caused by two compensatory substitutions fixed together in the same haplotype, which also predicts two sub-models. In the first, GC and AU represent both the highest fitness peaks, whereas AC and GU represent the lowest ones, and in another sub-model, GC, AU, GU and AC are the stepping stones from highest to lowest fitness peaks.

In mammals, most of the compensatory changes are Watson–Crick pairs (AU ↔ GC), other changes consisting of intermediate stage switches with lower fitness (e.g., AU -> AC and GC -> GU) [10,26]. However, the rupture of a pairing may be compensated by epistasis enhancing another pair in the stem (AU -> GC which has a stronger bond), the addition of an extra pair in D- and T-arms, changes of multiple interactions in stems and substitutions involving synergies between stem and loop structures [25]. About 10% and even up to 50% of the substitutions in mammals’ tRNAs are from an epistatic origin [25]. The fitness of stem base-pairs in *Hyalella* tRNAs presumably increases since most of FCBCs are AU -> GC and UA -> CG as described for humans and other mammals [10,25,79]. The opposite FCBCs (GC -> AU and CG -> UA) are also present but in much lower frequencies, as described in cetaceans [10]. On the other hand, we found very few wobble GU pairings in FCBCs in *Hyalella* stems and even lower AC pairings that have presumably even lower fitness. The pattern of HCBCs in *Hyalella* revealed the prevalence of changes toward wobble GU rather than Watson–Crick GC pairs, though the other Watson–Crick AU pairings were present at lower frequencies. Surprisingly, the most frequent HCBCs were A -> G vs. U (~33%) which are supposed to reduce the pairing’s fitness since bond strength is lower. Finally, mismatches may reduce the pairing’s fitness in stems, although their frequencies were lower than FCBCs and HCBCs. However, some mismatches are conserved in all *Hyalella* species studied such as the last pairing of the anti-codon stem of tRNA-I (UU, UC, CC). Thus, it can be hypothesized that other compensatory mechanisms may be acting as evolutionary constraints. The remainder four pairings in the anti-codon stem of tRNA-I are Watson–Crick AU and GC pairs that could compensate the mismatch as suggested previously [79]. In addition, shorter stems in D- and T-arms composed of two to three pairs are mostly formed with Watson–Crick pairs, particularly GC pairings, since they have stronger bonds. This hypothesis could explain why GC pairs are conserved in many short D- and T-arms across *Hyalella* species (e.g., tRNA-N, tRNA-I, tRNA-K, tRNA-L1 and tRNA-L2). The pattern of compensatory base changes in *Hyalella* described above is compatible with the hypothesis of “isolated fitness peaks” [26]. This can be deduced by (i) the presence of Watson–Crick switches, mostly FCBCs AU -> GC, caused by two compensatory substitutions fixed together in the same haplotype, (ii) a higher number of HCBCs GU pairs (wobble pairs with lower fitness than Watson–Crick pairs) and (iii) the residual detection of AC pairs with the lowest fitness. This hypothesis suggests that compensatory mutations in stems in *Hyalella* tRNAs navigate low fitness valleys as previously described in mitochondrial tRNA stem regions in 83 mammalian species [26]. The alternative “ascending continuous fitness ridges” hypothesis could also be operating if high frequencies of wobble GU pairings in HCBCs are under selection. However, this hypothesis would require that Watson–Crick switches occur independently [26].

Interestingly, the hierarchical clustering based on CBC occurrence across species is very consistent with the *Hyalella* phylogeny based on the DNA sequences of the 13 PCGs. Our results suggest that that fixation of tRNA CBCs can be used to infer the species evolutionary history, similarly as it was proposed for rRNA internal transcribed spacer sequences (ITS). For these regions, the presence of CBCs in secondary structures has been used as a proxy for species divergence [80]. Switches between AU and GC Watson–Crick nucleotide pairs at complementary sites of mitochondrial tRNAs occur 30–40 times more slowly than pairs of neutral substitutions in mammals [26]. AC and GU intermediates have been shown to involve a deep fitness valley. Therefore, they are negatively selected, implying that most CBCs could have been derived from the simultaneous fixation of two mutations that are individually deleterious [26]. The low probability of these events and their high fixation index could explain the phylogenetic signal contained in the *Hyalella* tRNA CBC data.

### 4.4. Selection in Protein-Coding Genes

Selection analyses show that negative (purifying) selection generally dominates mitochondrial genome evolution due to its importance in cellular respiration [71,81,82]. However, positive selection signatures can be detected in certain codons of mitochondrial *Hyalella* protein-coding genes. Our results agree with the numerous studies [81,83,84] showing higher functional constraint in the genes coding for COX proteins than in other mitochondrial genes. Cytochrome oxidase (*cox*) and cytochrome b (*cob*) showed the most conserved pattern, along with NADH dehydrogenase genes *nad3* and *nad4L*. The less conserved gene was *atp8*, concordant with previous analyses [2]. *Atp6*, *nad4* and *nad5* genes were more variable, with multiple codon sites subjected to positive selection in at least two analyzed tree-branches. The gene *nad5* also shows selection by the site analysis of CodeML (Model M8), even if not fully supported by the Bayes empirical Bayes test (<0.9 posterior probability). 

Positive selection was detected in particular positions of 8 of the 13 candidate genes in the branch-site model analyses using branch 1, corresponding to all *Hyalella* species estimated to be at least 25 my [33]. This result suggests a role in the adaptation of this amphipod group related to transition from marine or brackish to freshwater habitat conditions [85]. Interestingly, when branch 2 is defined as foreground (corresponding to a contrast between the mostly low-altitude *Hyalella* taxa versus species at high elevations), we found evidence for positive selection for some amino acids coded by the genes *atp6*, *nad1*, *nad2*, *nad4* and *nad5*. On the other hand, the analysis of more derived branches in the phylogeny corresponding to Andean Altiplano *Hyalella* lineages (i.e., branches 3, 4 and 5; Figure 1) reported few or no sites under selection. These selection results within *Hyalella* lineages may reflect recurrent adaptations to hypoxic conditions concomitant to the colonization of the high-altitude Andean lacustrine habitats.

TREESAAP results mostly agree with these findings (Figure 7). The majority of the amino acid sites in the PCGs showed a *Z* value close to zero or slightly negative. The PCGs with the higher number of sites above the threshold value were *atp6*, *atp8*, *nad4* and *nad5*. Moreover, *atp8* and *nad5* presented the higher number of physicochemical features (category 7 or 8) with a positive *Z*-score. 

## 5. Conclusions

In summary, (a) the pattern of compensatory mutations in *Hyalella* mitochondrial tRNA stems can be compatible with the isolated fitness peaks hypothesis and (b) the signatures of positive selection found in the *Hyalella* mitochondrial protein-coding genes indicate that these freshwater amphipods may have tuned their energy metabolism to adapt to novel habitats, in particular to freshwater and low atmospheric pressure and hypoxic conditions present at high altitude in the Andean Altiplano.

## Supplementary Materials

The following are available online at https://www.mdpi.com/2073-4425/12/2/292/s1, Table S1: Details on the *Hyalella* and other amphipod species used in this study with their corresponding GenBank accession numbers, Table S2: Details of the *Hyalella* mitogenomes examined. Mitogenome length (“*” indicates an incomplete mitogenome). Length of PCGs, tRNAs and rRNAs are indicated, Table S3: List of average nucleotide composition (A+T frequency), AT-skew and GC-skew for stems and loops of each tRNA sequence for the South American *Hyalella* species plus *H. azteca* from North America, Table S4: List of start and stop codons for the mitochondrial PCGs of South American *Hyalella* and the North American species *H. Azteca*, Table S5: List of the anti-codon sequence for each tRNA of the South American *Hyalella* species plus *H. azteca* from North America, Table S6: List of nucleotide changes found in tRNA sequences split by stems and loops for each tRNA of the South American *Hyalella* species plus *H. azteca* from North America. The first column for each tRNA indicates the position in the alignment, Table S7: Selective pressure analyses of *Hyalella* mitochondrial PCGs. Results correspond to the branch-site evolutionary analysis for positive selection using CodeML. The number of sites under positive selection with posterior probabilities > 95% are reported, Figure S1: Gene order comparison between the ancestral pancrustacean and the South American *Hyalella* mitogenomes. Shifts accounting for the observed differences between the two genome arrangements obtained with CREx are also shown, Figure S2: Heatmap of the average amino acid composition frequencies (top panel) and standard deviation (bottom panel) across *Hyalella* species, Figure S3: Heatmap of the average codon frequencies per thousand codons (top panel) and codon fraction per amino acid (bottom panel) across *Hyalella* species, Figure S4: Plot relating codon usages (ENC and MILC) vs. GC frequencies of third codon sites at fourfold degenerated amino acids at the gene level (top panel) and by coding strand (bottom panel), Figure S5: Mitochondrial gene order in four different amphipods: the whole Metacrangonyctidae family, *Parhyale hawaiensis*, *Pseudoniphargus daviui* and *Platorchestia*. Genes located above the central line correspond to genes coded on the plus-strand, whereas those coded on the minus-strand appear below. Blocks in grey correspond to non-coding segments attributed to control regions.
