# Supplementary material for: Comparative Mitogenomics in Hyalella (Amphipoda: Crustacea)
Source: Genes (Basel). 2021 Feb 19;12(2):292. doi: 10.3390/genes12020292 (PMC7923271; doi:10.3390/genes12020292)
Supplement: Supplementary file 1 [file genes-12-00292-s001.zip › FigS1.pdf]

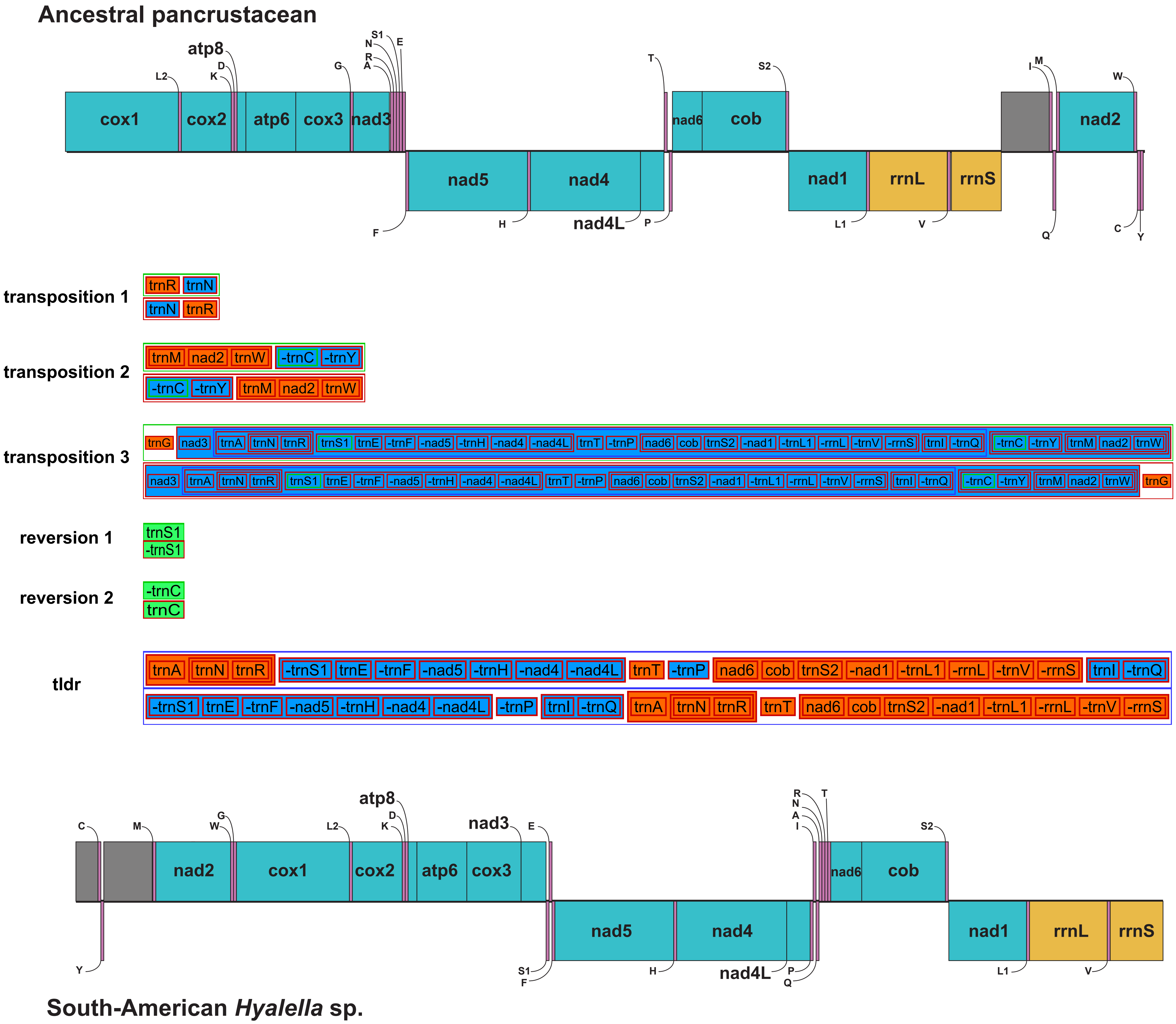
Ancestral pancrustacean
cox1
L2
cox2
atp8
K
D
atp6
cox3
G
nad3
R
A
N
S1
E
F
nad5
H
nad4
nad4L
P
T
nad6
cob
S2
nad1
L1
rrnL
V
rrnS
I
M
Q
nad2
W
C
Y
transposition 1
trnR trnN
trnN trnR
transposition 2
trnM nad2 trnW -trnC -trnY
-trnC -trnY trnM nad2 trnW
transposition 3
trnG nad3 trnA trnN trnR trnS1 trnE -trnF -nad5 -trnH -nad4 -nad4L trnT -trnP nad6 cob trnS2 -nad1 -trnL1 -rrnL -trnV -rrnS trnI -trnQ -trnC -trnY trnM nad2 trnW
nad3 trnA trnN trnR trnS1 trnE -trnF -nad5 -trnH -nad4 -nad4L trnT -trnP nad6 cob trnS2 -nad1 -trnL1 -rrnL -trnV -rrnS trnI -trnQ -trnC -trnY trnM nad2 trnW trnG
reversion 1
trnS1
-trnS1
reversion 2
-trnC
trnC
tldr
trnA trnN trnR -trnS1 trnE -trnF -nad5 -trnH -nad4 -nad4L trnT -trnP nad6 cob trnS2 -nad1 -trnL1 -rrnL -trnV -rrnS trnI -trnQ
-trnS1 trnE -trnF -nad5 -trnH -nad4 -nad4L -trnP trnI -trnQ trnA trnN trnR trnT nad6 cob trnS2 -nad1 -trnL1 -rrnL -trnV -rrnS
C
M
G
W
L2
atp8
D
K
nad3
E
R
N
A
I
T
S2
nad2
cox1
cox2
atp6
cox3
nad6
cob
Y
S1
F
nad5
H
nad4
nad4L
P
Q
nad1
L1
rrnL
V
rrnS
South-American *Hyalella* sp.
