# Supplementary figures and images for: Comparative Mitogenomics in Hyalella (Amphipoda: Crustacea)
Source: Genes (Basel). 2021 Feb 19;12(2):292. doi: 10.3390/genes12020292 (PMC7923271; doi:10.3390/genes12020292)

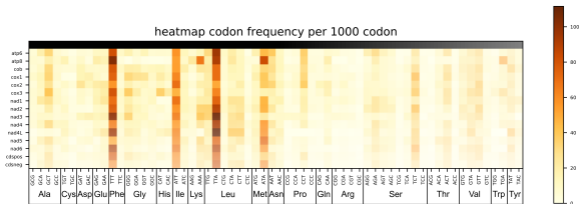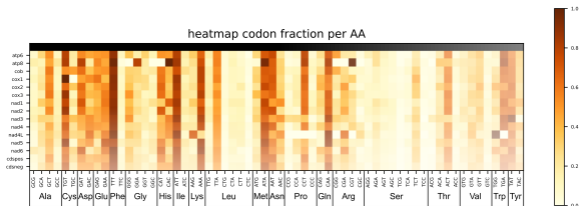

Supplement: Supplementary file 1 [file genes-12-00292-s001.zip › FigS3.pdf]

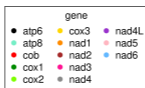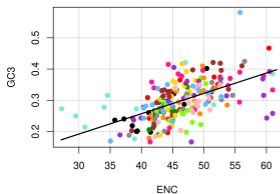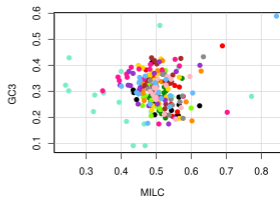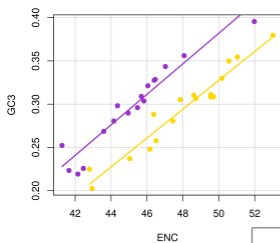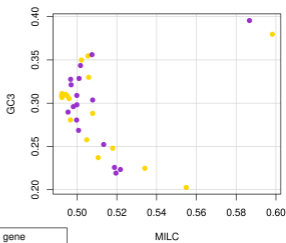

Supplement: Supplementary file 1 [file genes-12-00292-s001.zip › FigS4.pdf]

**Metacrangonyctidae**

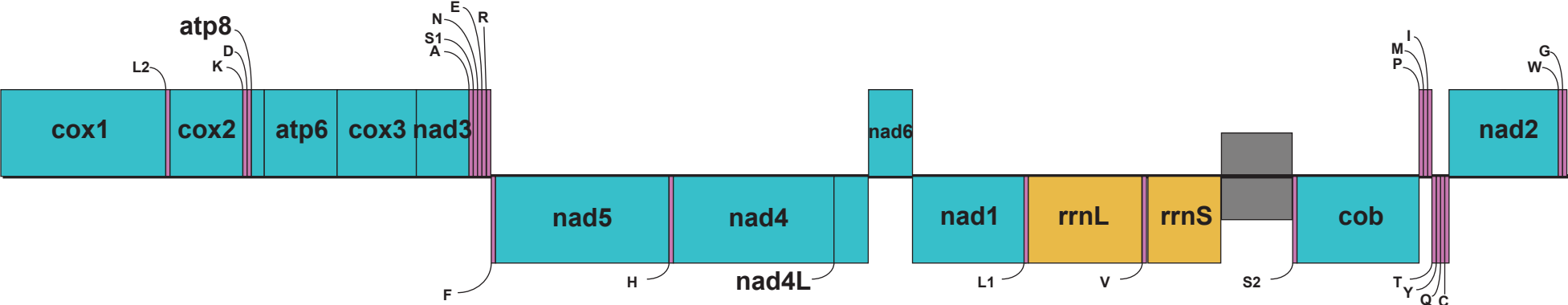

***Pseudoniphargus daviui***

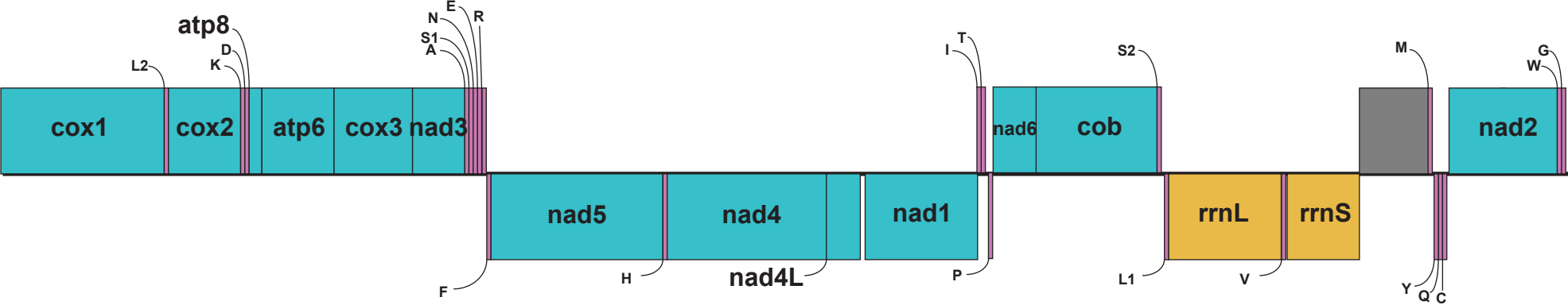

***Parhyale hawaiiensis***

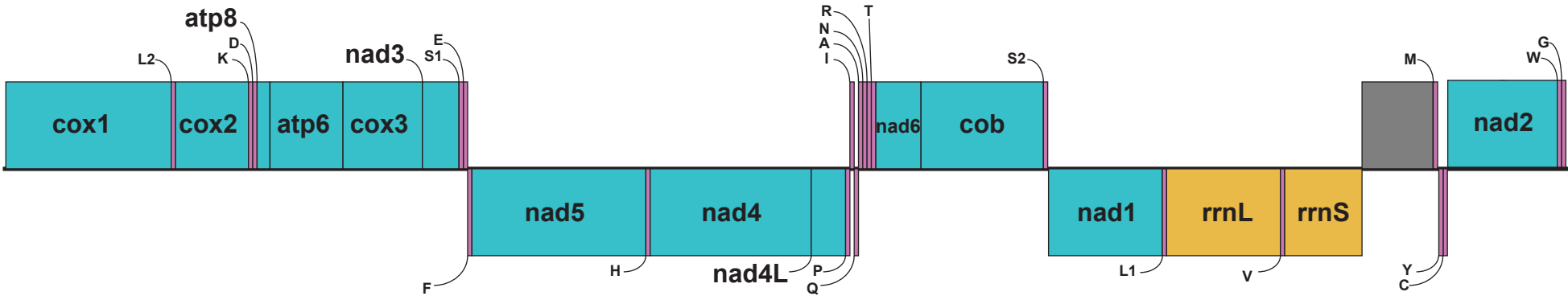

***Platorchestia* sp.**

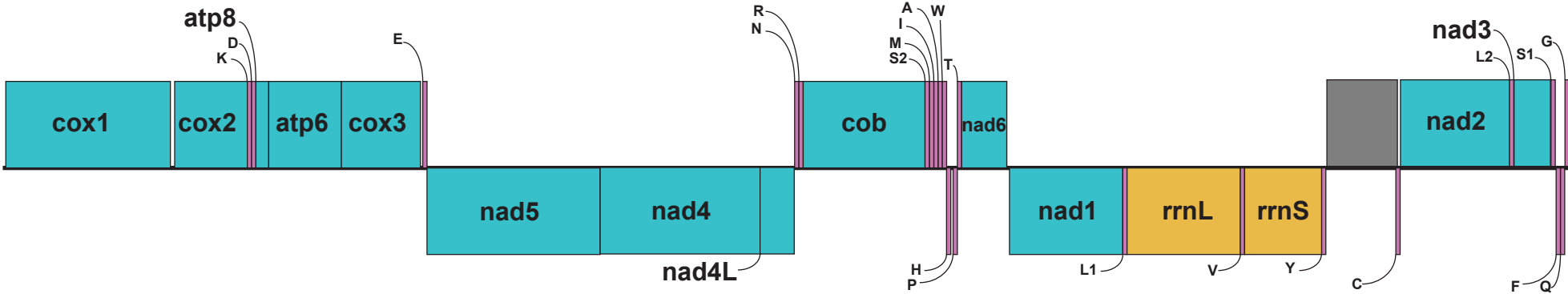

Supplement: Supplementary file 1 [file genes-12-00292-s001.zip › FigS5.pdf]
